# Toward a unifying taxonomy and definition for meditation

**DOI:** 10.3389/fpsyg.2013.00806

**Published:** 2013-11-20

**Authors:** Jonathan D. Nash, Andrew Newberg, Bhuvanesh Awasthi

**Affiliations:** ^1^Chiangmai, Thailand; ^2^Myrna Brind Center for Integrative Medicine, Thomas Jefferson University Hospital and Medical College Philadelphia, PA, USA; ^3^Waisman Center, University of Wisconsin-Madison Madison, WI, USA

**Keywords:** meditation, taxonomy, definition, contemplative traditions, contemplative neuroscience, cognition, affect, consciousness

## Abstract

One of the well-documented concerns confronting scholarly discourse about meditation is the plethora of semantic constructs and the lack of a unified definition and taxonomy. In recent years there have been several notable attempts to formulate new lexicons in order to define and categorize meditation methods. While these constructs have been useful and have encountered varying degrees of acceptance, they have also been subject to misinterpretation and debate, leaving the field devoid of a consensual paradigm. This paper attempts to influence this ongoing discussion by proposing two new models which hold the potential for enhanced scientific reliability and acceptance. Regarding the quest for a universally acceptable taxonomy, we suggest a paradigm shift away from the norm of fabricatIng new terminology from a first-person perspective. As an alternative, we propose a new taxonomic system based on the historically well-established and commonly accepted third-person paradigm of Affect and Cognition, borrowed, in part, from the psychological and cognitive sciences. With regard to the elusive definitional problem, we propose a model of meditation which clearly distinguishes “method” from “state” and is conceptualized as a dynamic process which is inclusive of six related but distinct stages. The overall goal is to provide researchers with a reliable nomenclature with which to categorize and classify diverse meditation methods, and a conceptual framework which can provide direction for their research and a theoretical basis for their findings.

## Analysis of the definitional and taxonomic issues

### Overview of the problems related to definition and description

In this section we consider various attempts to define meditation, and discuss the conceptual issues and the difficulties encountered.

The contemplative traditions and subsequent westernization have produced a proliferation of many disparate meditative practices utilizing different techniques and espousing different goals. For example, if one searchers the Yellow Pages Online Directories for major international cities one can find numerous listings under the heading “meditation” i.e., Los Angeles—156, Chicago—122, N.Y.C.—307, London—126, Sydney—139, and Stockholm—108 (as of August 1, 2013). It is quite apparent that “meditation” has become a generic term, used to describe a host of secular, spiritual, and/or religious contemplative activities; as well as becoming a synonym for many other more mundane cognitive functions such as contemplation, reflection, concentration, and terms such as ponder, ruminate, cogitate, and deliberate. Although convenient for everyday usage, the casual use of a generic term to group any activity or practice can cause obvious difficulties in communication due to false assumptions of similarity.

Neuroscientists and other meditation researchers have also floundered on this point by commonly using the generic term “meditation” to refer to a wide variety of disparate methods which “inevitably trivializes the practices themselves” (Lutz et al., 2007, p. 500). In addition there has been a tendency to mix-and-match different methods as if they were equivalent which has resulted in an unfortunate conflation of definition (Awasthi, 2013). This struggle to clearly define meditation is affirmed by Bond et al. (2009) who identified five commonly used definitional themes in their review of the meditation research literature. Currently, the scientific literature contains two popularly used definitions for meditation. One “camp” has defined meditation essentially as a family of mental training techniques (e.g., Cahn and Polich, 2006; Lutz et al., 2008b; Raffone and Srinivasan, 2010)—which we shall call the “method definition.” The other “camp” has defined meditation by reference to the enhanced experiential states or altered states of consciousness which arise from the use of these methods e.g., “pure consciousness^1^,” “absolute unitary being^2^,” and “non-dual awareness^3^”—which we shall call the “state definition.” It is obvious that difficulties in comparing research results are inevitable when investigators use two essentially different definitions to refer to the same term.

This conflation of definition is exemplified by recent commentary regarding attempts to classify Transcendental Meditation™ (Josipovic, 2010; Travis and Shear, 2010a,b), in which the authors use several conceptually distinct lexicons to argue their positions. There is a discussion as to whether “focused-attention” (FA)^4^, “open-monitoring” (OM)^5^, or “non-dual awareness” (NDA) are appropriate classifications for TM. However, FA and OM were formulated as theoretical categories of meditation techniques based on two different cognitive strategies employed during the method stage (Lutz et al., 2008b); whereas NDA was devised to describe a particular enhanced state of awareness. In this case, one author was objecting to the appropriateness of using a certain definition of method, while the other author was using a definition based on the resultant state. Adding to the perplexity, Travis asserted that TM should not be classified into any of the aforementioned lexicons. He offered a fourth category based on an entirely different notion—“automatic self-transcending”; a hypothetical proposal of what happens when there is a shift from one state of consciousness to another.

On the other side of the coin, several authors have stressed the importance of making an explicit distinction between method and state, whereas the state is considered to be the causal result of the successful application of the method (West, 1987b; Koshikawa and Ichii, 1996; Lutz et al., 2007; Awasthi, 2013).

Contributing to this definitional problem is the ubiquitous paucity of information given when researchers describe methods of meditation chosen for study (Lutz et al., 2007; Ospina et al., 2007). Researchers typically select their meditator-subjects from a locally available group of individuals who have been engaged in a particular method of practice; and then identify the method by its name and tradition as if this was sufficient. Occasionally a general description is offered, but mostly there is a failure to account for and report many of the salient particularities of the chosen method—such as non-verbal vs. verbal, eyes open vs. closed, natural vs. regulated breathing, static vs. kinetic body movements, etc. Although there have been some attempts to describe meditation methods by utilizing certain salient features (Koshikawa and Ichii, 1996; Lutz et al., 2007; Ospina et al., 2007), as of yet neuroscientists have not employed a standardized protocol to describe the meditation methods they are studying. We contend that this omission is a significant impediment to the advancement of this field given that any, and perhaps all, of the salient features of a given technique may affect neurobiological findings (see section The Taxonomic Keys).

To summarize, even a casual review of the literature reveals a frequently occurring theme lamenting the lack of a standardized approach to define “meditation,” and the challenge this poses for researchers (West, 1987b; Taylor, 1997; Lutz et al., 2007; Ospina et al., 2007).

We concur that it is counter-productive if the term “meditation” is employed in a generic or non-descript way, and if there is an unclear distinction between the notions of method and state. We posit that these definitional issues may have contributed to the confusing, contradictory, and inconsistent findings that have emerged from the field of contemplative neuroscience. As we discuss later on in this paper, since method and state demonstrate different neurobiological correlates, researchers need to recognize what they are measuring during a given meditation session and carefully distinguish method from state. For example, the effects on resting state functional connectivity (the Default Mode Network) in various meditation styles, often reported in the literature with seemingly inconclusive/mixed findings (Awasthi, 2013), may be attributed to this conflation of definition.

### Overview of the problems related to taxonomy

*A well-conceived and useful taxonomy has the power to frame all theoretical considerations of a particular field of study. It is natural, and in fact historical, for scientists and philosophers to desire to segregate and classify the things and processes of this world.* (Ereshefsky, 2000, p. 50–51).

In this section we review and analyze various attempts to devise taxonomic categories for meditation methods, an approach quite similar to Overview of The Problems Related to Definition and Description above. In some ways this can be viewed as an artificial distinction, in that the issues and particulars of taxonomy often overlap with those of definition, especially from an historical perspective. However, we feel that certain important distinctions warrant separate consideration.

The struggle to formulate a universally acceptable taxonomy of meditation has been well-documented and is exemplified by the work of Ospina et al. (2007). Their Evidence Report: Meditation Practices for Health: State of the Research was undoubtedly the largest, most ambitious, and most comprehensive survey of meditation research ever undertaken. It included an analysis of over 1000 published papers and references. They attempted to group over 30 meditation methods into “five broad categories” using an empirical taxonomic approach: Mantra, Mindfulness, Tai Chi, Qigong, and Yoga. However, this nomenclature appears to have been contrived without an overarching theoretical framework. The first category refers to the conceptual focus of attention, the second refers to the cognitive strategy employed, and the others were based on three different traditional forms of practice. It is understandable then that their effort to construct a taxonomy was frustrated “given the variety of the practices and the fact that some are single entities … while others are broad categories that encompass a variety of different techniques.” They concluded that it was “impossible to select components that might be considered universal or supplemental across practices” (p. 2–3); that “characterization of the universal or supplemental components of meditation practices was precluded by the theoretical and terminological heterogeneity among practices” (p. 5); and that since the term “meditation is an umbrella term … this lack of specificity … precludes developing an exhaustive taxonomy of meditation practices. (p. 10).

Currently, neuroscientists and other researchers still do not have an overarching, consensual framework with which to understand and study this highly specialized form of mental “exercise” and its many different forms. It has occurred to us that this situation, with its robust semantic difficulties, is somewhat similar to the conundrum that Linnaeas faced in the 1700's.

*“Three hundred years ago biological taxonomy was a chaotic discipline marked by mis-communication and misunderstanding. Biologists disagreed on the categories of classification, how to assign taxa to those categories, and even how to name taxa. Fortunately for biology, Linnaeas (attempted to) … bring order to taxonomy. The system he introduced offered clear and simple rules for constructing classifications. It also contained rules of nomenclature that greatly enhanced the ability of biologists to communicate.”* (Ereshefsky, 2000, p. 1).

Common reasons given for the taxonomic difficulties we face today are the complexity and diversity of meditation techniques and the ineffable nature of the meditative state. However, we posit that many of these difficulties can also be attributed to the exclusive use of first-person constructs, and the limitations of the taxonomic methods that have been employed.

The first point is illustrated by the plethora of lexicons that have been devised to classify meditation methods over the last 40 or so years. Early proposals [as reviewed by West (1987a)] used terms such as “concentrative vs. opening-up” (Ornstein), “concentrative vs. insight” (Goleman), and “wide angle lens attention vs. zoom lens attention” (Shapiro); and recently new terminology has been introduced such as “open presence^6^” (6); the “active” and “passive” approaches (Newberg et al., 2001); and the previously mentioned “focused attention,” “open monitoring,” “non-dual awareness,” and “automatic self-transcending.” These first-person semantic constructs have undoubtedly been helpful, but their use as a basis for taxonomy is not without problem. We argue that this strategy has contributed to misunderstanding, ambiguity, and confusion, and has been an impediment toward consensus. This is not to minimize or reject the value of first-person accounts as important phenomenological data for meditation research (Pekala, 1987). They have certainly been necessary for such tasks as the recording of “peak experience” (Newberg and d'Aquili, 2001) and “transcendence” (Travis and Pearson, 2000; Travis et al., 2002), or in the broader study of consciousness (Wallace, 2000; Costall, 2006). However, such accounts can be somewhat unreliable as they depend on the accurate recording of the mental states of the subject and the proper accounting of a host of varied external and internal factors. Hence, first-person accounts lack the stability and consistency required to provide a stand-alone, reliable foundation for scientific research. Although first-person reports are potentially useful as data, first-person constructs are theoretically and semantically ill-suited for the formulation of orthogonal taxonomic domains. The difficulties facing the scientific use of first person accounts has been well known to cognitive scientists since the early 20th Century critique of “introspectionism,” but for some reasons this seems to have escaped the scholars and neuroscientists attempting to establish scientifically usable categories for meditation taxonomies (Dreyfus, 2013, pers. commun.).

We have concluded that an alternative approach is required. We contend that the use of a widely accepted third-person paradigm offers the potential for a clearer semantic distinction and a more reliable platform/framework for research, as opposed to the use of unique but ambiguous first-person categories, however enticing those may be.

In addition, prevailing taxonomies of meditation have employed a “piecemeal” approach similar to classical “phenetic” taxonomic philosophy (Ereshefsky, 2000). That is, they have attempted to segregate meditation methods based on observable characteristics or features e.g., grouping all methods that use a mantra under one rubric; or trying to group methods according to the particular cognitive strategy employed. We argue that while such particulars may be suitable for lower order classifications of meditation, they are not sufficient for the formulation of an over-arching system of orthogonal Domains. There are simply too many different methods, features, nuances, and traditions to permit higher-order categorization based solely on characteristics (Ospina et al., 2007). While taxonomic pheneticism may be effective for biological systems, we propose that an alternative taxonomic approach may prove to be more suitable for the classification of meditation.

#### Summary

We propose to use the above analysis of the extant problems and their root causes to devise a new definition and taxonomy for meditation. A multi-faceted approach will include: defining meditation as a dynamic process with separate stages that unfold over time; a new taxonomic system which uses a well-established third-person paradigm, in conjunction with some necessary first-person perspectives, to formulate three Linnaean-type overarching Domains; and describing and segregating methods within each Domain according to a table of taxonomic keys.

## A proposal for a new definition and taxonomy

Given the extant problems of definition and classification, how then can we proceed to formulate a more effective descriptive model for the discussion and study of meditation?

We proceed by defining the difference(s) between method and state, and then propose a model which describes how they interact as distinct stages of a dynamic process.

### Definition

#### Rationale

As previously discussed, meditation has been typically defined in one of two ways—as either a family of mental training techniques (the “method definition”), or in relation to the particular altered states of consciousness that arise from the implementation of the technique (the “state definition”). We address this potentially confusing duality by proposing a model of meditation which is inclusive of both method and state. In this paradigm, method and state are viewed as separate stages in a dynamic process which unfolds over time. The method is considered to be a potentially facilitative tool and the state is the causally-related intended result. As such, we support the premise that researchers need to recognize and carefully segregate these two stages so as not to confound “the neural correlates of the meditation techniques that are used to get to particular ‘states’ of consciousness, with the correlates of the ‘states’ themselves” (Josipovic, 2010).

#### A new definitional model of meditation as a dynamic process

The various stages of this dynamic process can be represented by a standard flow diagram (see Illustration 1) which depicts their relationship to each other as a meditation session progresses, and helps to explain how the model works. A given meditation session is defined as the time allocated by the meditator to the engagement of the process; whereby the meditator starts from a mundane state of alert/waking consciousness, moves through the specific stages of the process over time, and then returns to that same state of waking consciousness. This does not infer that nothing has changed with regard to trait or plasticity, but rather that one begins and ends in essentially the same state of consciousness.

This idea of considering meditation as a series of stages has been proposed by others, most recently by Tang et al. (2012) who advanced the idea of three stages of meditation practice. In this model, meditation is codified into six stages. We also suggest that there are important neurobiological correlates associated with most of these stages.

***Normal (N).*** This is the pre-meditative stage of normal waking/resting consciousness. This is the baseline state where one is mentally preoccupied with the mundane thoughts, feelings, and activities of daily life; and which can be distinguished from other states of consciousness by certain defining neurobiological correlates (Lou et al., 1999; Vaitl et al., 2005; Lutz et al., 2007). Interestingly, this is an area that has received some attention in the neuroscientific literature. Specifically, this subjective state appears to be linked to what is currently regarded as the “default network” in the brain, first described by Raichle et al. (2001). The structures that tend to be involved in this resting or baseline state of the brain play a role in how we ultimately utilize the brain for various cognitive and affective tasks. The default mode network essentially allows the brain to be poised to be able to react to various stimuli or activities. There are several studies that have suggested that the resting brain is actually altered by meditation practices. Studies of long term meditators have revealed differences in the baseline structure and function of the brain (Lazar et al., 2005; Pagnoni and Cekic, 2007; Pagnoni, 2012) and, more specifically, in the default network (Jang et al., 2011; Berkovich-Ohana et al., 2012). Furthermore, longitudinal studies have shown that the resting state of the brain can be altered by employing a regular meditation practice. Thus, the normal resting state of the brain is still a highly relevant concept in the context of meditation techniques and their effects.

***Intention to begin (IB).*** This is the willful intention to initiate the process, which manifests in the actions taken to implement the subsequent stages of the process. One can speculate that this intention could be sustained through the preliminary stage until the actual engagement of the Method. This is a key denotation in our model because it highlights the importance of volition in the engagement of the meditation process. In this paradigm meditation is considered as a conscious and willful act unaided by any extrinsic mind-altering substances, whereby the meditator exercises personal control to employ a particular method. Therefore, we also exclude the occasional transcendent experience brought on by a serendipitous “special moment,” as well as the experience of being hypnotized by someone else through the power of suggestion.

Initial models of meditation have indicated that this stage has a neurophysiological correlate, most likely in the prefrontal cortex (PFC) which is involved in the initiation of willful behaviors. Early studies demonstrated increased activity in the PFC during many different types of meditation practices. Brain imaging studies suggest that willful acts and tasks that require sustained attention are initiated via activity in the PFC, particularly in the right hemisphere (Posner and Petersen, 1990; Frith et al., 1991; Pardo et al., 1991; Ingvar, 1994). The cingulate gyrus has also been shown to be involved in focusing attention, most likely in conjunction with the PFC (Vogt et al., 1992). Since meditation methods often require intense focus of attention, it seems appropriate that the initiation of the meditation process begins with activation of the PFC (particularly the right) as well as the cingulate gyrus. This notion is supported by the increased activity observed in these regions in several of the brain imaging studies of volitional types of meditation (Herzog et al., 1990--1991; Lazar et al., 2000). In a study of Tibetan Buddhist meditators, there was increased activity in the PFC bilaterally (greater on the right) and the cingulate gyrus during meditation (Newberg et al., 2001). Therefore, meditation appears to start by activating those areas of the cortex associated with the will or intent to clear the mind of thoughts or to focus on an object.

***Preliminaries (P).*** This is a preparatory phase in which a particular setting and certain rituals may be employed to set the “proper tone” for the meditation session. This could consist of simply going into a specially designated room, turning off the lights, and sitting comfortably; and/or using a special pillow or cushion, lighting incense and/or candles, donning a special shawl, playing certain types of music, etc.

Rituals have certainly been found to have a substantial impact on brain function, in part through rhythmic or repetitive elements. Thus, specific music, phrases, or objects for which the brain is familiar, may initiate those processes that will proceed in the method stage. For example, a number of studies have suggested that music affects the brain (Satoh et al., 2003; Saito et al., 2006; Eldar et al., 2007) particularly in the limbic or emotional centers of the brain (Newberg et al., 2010). While there is less evidence for the neurophysiological effects of specific preparatory processes, one might speculate that activation of the amygdala to signify that an important activity is about to occur may help to move the brain from the default network processes to the purposeful processes of the method stage involving the PFC (Newberg and Iversen, 2003).

***Method (M).*** Methods, in a general sense, can be thought of as procedures or techniques that are employed in order to do or accomplish something. With regard to meditation, methods have been defined as “a family of complex emotional and attentional regulatory training regimes developed for various ends, including the cultivation of well-being and emotional balance” (Lutz et al., 2008b, p. 163). Typically, one would use a training regime in an attempt to acquire a particular skill for a particular purpose, through practice and instruction over a period of time. When we apply this notion to meditative practices we see that the method is used to develop the skills to regulate (control or direct) the mental faculties of attention and emotion. We will return to these notions of attention (cognition) and emotion (affect) later on in this paper.

Meditation methods typically purport to yield both immediate and long-term outcomes: the attainment of certain altered states of consciousness during a given meditation session—often referred to as the “state” effect (Lehmann et al., 2001; Lutz et al., 2007); and that continued practice can lead to the realization of other goals or benefits that relate to a person's overall approach, skill set, or perspective on life—often referred to as the “trait” effect (Cahn and Polich, 2006; Lutz et al., 2007; Raffone and Srinivasan, 2010). For the purposes of this proposal, we have chosen to focus on that which transpires during a given meditation session—the state effect.

In this model we consider the Method to be simply the prescribed set of instructions that the meditator employs during a meditation session. These instructions are usually imparted by a teacher, or another form of didactic medium such as books, videos, tapes, etc. Methods invariably contain various cognitive strategies variously described as: concentration, mindfulness, “focused awareness,” passive observation, “open-monitoring,” memorization and repetition (of certain words, phrases, or narratives), self-inquiry, contemplation, imagination and visualization, and perception (of sounds, lights, and bodily sensations). Also, the mental functions of metacognition and control (Koriat, 2007) play an important role in the meditation process (Hasenkamp et al., 2012; Pagnoni, 2012). The use of metacognitive awareness and control of mind-wandering allows the meditator to be aware of the stages of the process and stay on task.

In addition, we think it is important to recognize that the Method itself only holds a potential for success. One cannot assume that the employment of a particular method is a guarantee of efficacy because there are a host of extrinsic and intrinsic factors that may obviate the desired shift in consciousness e.g., predispositions, biases, the presence of certain drugs or intoxicants, mental disorders, inability to concentrate, external distractions or interruptions, etc. For these reasons we utilize the notion of the “intention” of the method in the formulation of our taxonomic nomenclature (see sections Determination of the Functional Essence of Meditation Methods and Formulation of a Taxonomic Nomenclature for Meditation Methods).

***Enhanced mental state (EMS).*** This is the causal result of the successful application of the Method—an altered state of consciousness, commonly referred to as the meditative state. It is usually accompanied by subjective first-person reports of a shift in consciousness to a different and more “profound” state such as: an enhanced sense of well-being, focus, calm, detachment, insight, affect, bliss, emptiness, etc. EMS has been shown to have neurobiological correlates distinct from the normal resting state and other mundane states of human consciousness (Travis and Pearson, 2000; Newberg and d'Aquili, 2001; Vaitl et al., 2005; Bærentsen et al., 2009). We will consider the evidence and theory supporting this distinction in the following section. EMS may manifest as a fleeting, momentary state (as typically reported by novice practitioners), or may be sustained for considerable periods of time (as typically reported by advanced/experienced practitioners).

Our model articulates three primary types of EMS which will be discussed in detail in section Formulation of a Taxonomic Nomenclature for Resultant States.

***Intention to finish (IF).*** Is the termination of the meditation session in which the practitioner elects to end the process and return to a mundane state of waking consciousness.

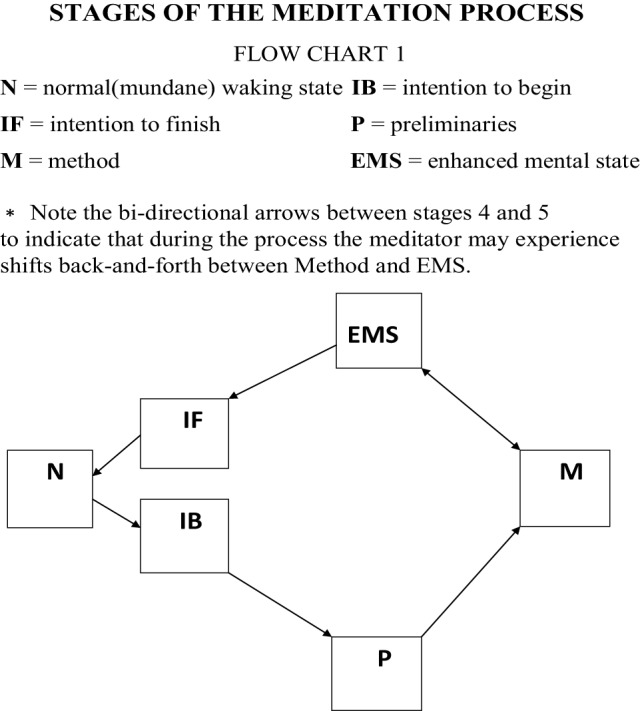



#### Other possibilities

Our definitional model of meditation as a dynamic process is intended to be inclusive of several different possibilities, and defines meditation as an engagement of the process with the intent of attaining an EMS. Under certain circumstances, even though a practitioner may not engage all the stages of the process, this would still be considered to be “meditation” according to our model e.g., in the case of a novice practitioner who engages the first three stages but is not successful in attaining EMS; the practitioner who is disturbed during the Preliminary or Method stages and quits due to extrinsic factors outside of his/her control; or in the case of an experienced practitioner who no longer needs the “training wheels” *per se*, and is capable of going directly to an EMS at will without the aid of the Preliminary or Method stages.

### Taxonomy

#### The rationale for a new taxonomic model

We have attempted to avoid the problems encountered by previous efforts by employing alternatives to the prevalent first-person/phenetic strategies.

***A third-person approach.*** As previously noted, researchers and scholars have typically relied on first-person perspectives and their own linguistic ingenuity to fabricate original categories and new terminology. We have argued that such first-person attempts often lack scientific reliability and are thus easy targets for criticism and disagreement when used in such a manner; and that an accepted third-person construct could provide a more reliable semantic paradigm. Since meditation can be considered to be a mental phenomenon, we decided to look to the fields of Psychology and Cognitive Science for an appropriate third-person paradigm that could serve as the cornerstone of a new taxonomic nomenclature. We chose the historically well-founded and commonly accepted paradigm of Affect and Cognition (although this is admittedly an arbitrary decision and other third-person paradigms may also be suitable). We have made this choice fully recognizing that modern theories speak to the inter-relationship of Affect and Cognition and do not consider them to be totally separate and distinct faculties (Forgas, 2008). Therefore, when we refer to affective or cognitive states in this paper, we are referring to the predominant subjective and neurobiological correlates of those states, and are not assuming that such states are in some way “totally pure”—see Formulation of a Taxonomic Nomenclature for Resultant States. For the purposes of this paper we reduce to commonly accepted definitions; whereas Affect includes emotions and feelings, and Cognition includes a multitude of mental processes associated with thinking, including (but not limited to): learning, reasoning, observing, perceiving, remembering, imagining, processing information, and acquiring knowledge.

***An essentialist approach.*** In addition, we attempt to avoid the problems created by classifying meditation methods solely according to their particular characteristics or features (the aforementioned “phenetic” approach), which we have argued is problematic for such a complex phenomenon. We have adopted an “essentialist”-type approach similar to the Aristotelian and Linnaean schools of taxonomy (Ereshefsky, 2000), which requires that we first determine the functional essence of whatever it is that we want to taxonomize. We attempt to describe this functional essence not in a philosophical way, but rather in a way that would be useful and measurable for researchers.

#### Determination of the functional essence of meditation methods

Based on our review of the various contemplative traditions and the published research in this field, we utilize the following properties and assumptions regarding meditation methods:

- We assume that meditation methods have been derived for specific purposes and goals.
- There is mention of ultimate, long-term benefits and goals (the aforementioned “trait” effect) such as: the attainment of enhanced attentional and emotional acumen, purification of the mind and/or the heart, stress-reduction, a greater sense of well-being, attainment of wisdom, equanimity, compassion, liberation, enlightenment, etc.
- In order to accomplish these ultimate goals, meditation methods utilize techniques that have been designed to accomplish a more immediate goal—to facilitate a shift from the normal state of consciousness to an altered state of consciousness within a given meditation session—the aforementioned “state” effect. We have used the term EMS to describe this altered state.
- Methods will vary depending on their ultimate goal(s) and their particular techniques that have been designed to engender a targeted EMS relevant to that goal.
- The successful attainment of the targeted EMS ultimately depends on a host of extrinsic and intrinsic factors. With regard to the practitioner, one must account for such factors as experience and expertise, motivation, current state of mind and body, the presence of intoxicants or other drugs, etc. These intrinsic factors will undoubtedly affect the quality and experience of the EMS regardless of what the method purports or intends to accomplish.
- Therefore, we must consider the method to be just a facilitative tool, which only offers the potential, not a guarantee, for the attainment of immediate and ultimate goals.
- The ultimate goals are more challenging (if not impossible) to measure and evaluate given current scientific knowledge and instrumentation.

For these reasons, we have defined the functional essence of the method in terms of its intended immediate goal—the targeted EMS. That is, what it was designed to do during a given meditation session that could be measured in a laboratory setting. This idea that a given method can possess a specific intended or targeted outcome is supported in the literature by those who discuss the need to account for the aims, purposes, goals, and effects of particular meditation techniques (West, 1987b; Koshikawa and Ichii, 1996; Lehmann et al., 2001; Carter et al., 2005; Hankey, 2006; Lutz et al., 2007; Ospina et al., 2007). Since it is somewhat awkward to think of methods as having intention, for the purposes of this paradigm we use the idea of “directionality” to convey this notion of a targeted outcome. In this sense the EMS can be thought of as the immediate destination, and the Method as the set of instructions, or the map, of how to get there.

#### Formulation of a taxonomic nomenclature for meditation methods

We proceed by combining taxonomic essentialism with a third-person approach. By framing this notion of directionality within the aforementioned paradigm of Affect and Cognition, we have formulated a new taxonomic nomenclature consisting of three overarching Domains:

- **The Affective Domain** represents those methods which purport to engender an enhanced affective state (EAS) during the meditation session. These are typified by traditional methods such as the compassion and loving-kindness techniques of Tibetan and Theravada Buddhism. These methods would be classified as affective-directed methods (**ADM**).
- **The Null Domain** represents those methods which purport to create an enhanced empty state that is devoid of phenomenological content—a non-cognitive/non-affective state (NC/NA EMS). Such methods would be classified as null-directed methods (**NDM**), typified by such techniques as TM, Zen satori methods, and Yoga methods aimed at the dissolution of the sense of self.
- All remaining techniques (by default) fall to **the Cognitive Domain** and thus would be classified as cognitive-directed methods (**CDM**). These are typified by traditional methods such as *samatha* and *vipassana* and would include all those methods that purport to engender an enhanced cognitive state (ECS) i.e., one-pointedness, mindfulness, or insight.

The assignment of Domains to actual meditation methods is demonstrated by example in section Use of the Taxonomic Keys and Domains, Nine Examples.

#### Formulation of a taxonomic nomenclature for resultant states

Once we have devised a classification of methods based on directionality, we then formulate an appropriate nomenclature for the resultant states. In this taxonomic model, each type of method is causally related to one of the following three resultant states^a^(see Ill. #2 below). This hypothesis is based in part on the idea that enhanced states of cognition, affect, and emptiness demonstrate distinctly different and measurable subjective and neurobiological correlates (Travis and Pearson, 2000; Lehmann et al., 2001; Dagleish, 2004; Carter et al., 2005; Hankey, 2006; Lutz et al., 2007, 2008a; Davidson, 2010; Travis and Shear, 2010a; Josipovic et al., 2012; Leung et al., 2013).

***Enhanced cognitive state (ECS).*** Is defined as the resultant state of consciousness due to the successful employment of a CDM, in which the phenomenological content is primarily cognitive in nature. For example, we would classify the resultant state to be an ECS if a particular technique resulted in complete “one-pointedness”—“the maintained focus of attention on a single object” (Carter et al., 2005, p. 412). There is a significant body of research in support of this notion of an enhanced cognitive meditative state.

***Supportive neuroscientific findings.*** From a brain perspective, we would likely consider such a state as involving activity in one or more of the cortical areas of the brain that subserve higher cognitive processing such as areas of the brain that support verbal reasoning or abstract processes. A number of studies have reported increased functioning in the frontal lobes particularly the PFC in subjects performing a concentration-based cognitive directed meditation practice (Herzog et al., 1990--1991; Lou et al., 1999; Lazar et al., 2000). For example, Tibetan Buddhist meditation that incorporated concentration on a visual object demonstrated a number of complex changes including relatively increased cerebral blood flow (CBF) in the PFC and cingulate gyrus (Newberg et al., 2001). Another study found that Vipassana meditation activated the rostral anterior cingulate cortex (ACC) and the dorsal medial PFC in both hemispheres (Holzel et al., 2007). In addition these investigators found that Vipassana meditation might enhance cerebral activity in brain areas related to interoception and attention, such as the PFC, the right anterior insula and the right hippocampus (Holzel et al., 2008). Thus, these findings support the taxonomic approach that cognitive-directed meditation practices activate cortical areas involved in cognitive functions such as attention and abstract thought. Additional support for this notion can be found in studies evaluating differences in “trait” characteristics and task performance between experienced and less-experienced meditators; and several of these reports have shown that such cognitive-directed practices result in overall improved cognitive processing which can be related to site-specific cortical function (Valentine and Sweet, 1999; Carter et al., 2005; Chan and Woollacott, 2007; Pagnoni and Cekic, 2007; Moore and Malinowski, 2009; Hodgins and Adair, 2010; van den Hurk et al., 2010; Pagnoni, 2012).

***Enhanced affective state (EAS).*** Is defined as the resultant state of consciousness due to the successful employment of an ADM, in which the phenomenological content is primarily an emotion or feeling such as loving-kindness or compassion (so called matters of the “heart”). Lutz describes this as “the generation of a state in which an unconditional feeling of loving-kindness and compassion pervades the whole mind as a way of being, with no other consideration, or discursive thoughts” (2008a, p. 1) Although these are not considered as emotions by traditional Buddhist philosophy, they can be considered as affect when interpreted into Western/English “mental typologies” (Dreyfus, 2002), and many modern researchers have done so. There is a significant body of research in support of this notion of an enhanced affective meditative state.

***Supportive neuroscientific findings.*** Affect can be distinguished by distinct and measurable subjective and neurobiological correlates (Dagleish, 2004; Hanson and Mendius, 2009). Many modern researchers consider compassion/loving-kindness not only to be an expression of affect but have reported distinct neurophysiological correlates associated with this state (Lutz et al., 2008a; Davidson, 2010; Menezes et al., 2012; Leung et al., 2013; Mascaro et al., 2013). For example, Lutz et al. (2008a) reported distinctive activation of the limbic regions including the insula and cingulate cortices; right temporoparietal junction; and posterior superior temporal sulcus during Tibetan Buddhist compassion meditation. Davidson (2010) hypothesized that loving-kindness meditation activates circuits associated with positive affect including the ventral striatum, orbital frontal cortex, and dorsolateral regions of the PFC. Lutz et al. state that certain meditation methods can “regulate emotions associated with altered activation of the limbic system” (2008a, p. 2). Mascaro et al. (2013) reported increased neural activity in the IFG and the dmPFC during compassion meditation. Leung et al. (2013) reported increased gray matter volume in the right angular gyrus and right posterior parahippocampal gyrus in subjects performing loving-kindness meditation. Furthermore, there appears to be a distinct pattern of brain activity associated with meditation practices associated with an EAS vs. those associated with an ECS. For example, Carter et al. (2005) reported distinct differences of functional effects on the visual switching rivalry between one-pointedness meditation and compassion meditation, and Travis and Shear (2010a) reported different EEG patterns for compassion meditation vs. focused attention vs. TM.

***Enhanced non-cognitive/Non-affective state (NC/NA).*** Is defined as the resultant state of consciousness due to the successful employment of a NDM. This enhanced state is much more challenging to define as it infers the absence of affect and cognition—an empty state with no phenomenological content. This notion of emptiness has manifested in a host of semantic constructs derived from diverse spiritual/religious traditions and languages i.e., nirodha-samapatti (Pali), samadhi (Sanskrit), satori (Japanese), dzogchen (Tibetan). However, attempts to translate these terms into English have struggled to capture the essence of this ineffable and non-conceptual state of consciousness. As such, many different terms have evolved depending on cultural/religious belief systems, linguistic perspectives, and perceptions of the underlying ontology of meditation practice. The examples are numerous and include such ideas as: God Consciousness, Christ Consciousness, Buddha Consciousness, cosmic consciousness, pure consciousness, true-Self, non-Self, NDA, absolute unitary being; and other terms such as Formless, Void, emptiness, and undifferentiated “beingness” or “suchness.” We can also look to well-known Yogic teachers or Masters for their commentary. According to Sri Nisgaradatta Maharaj, there is a merging into a state of nothingness accompanied by a loss of sense of Self and duality (Powell, 1994); Osho describes samadhi as “no object in the mind, no content ……, not meditating upon something, but dropping everything (so that) not even a ripple arises in the lake of your consciousness.” (Osho, 2003); and Sri Ramana Maharshi states that “samadhi is the state in which the unbroken experience of existence is attained by the still mind” (Godman, 1985). For the purposes of this essay, we consider all these terms and descriptions to refer to the same state. There is a significant body of research in support of this notion of an enhanced non-cognitive/non-affective meditative state.

***Supportive neuroscientific findings.*** From a neurophysiological perspective, we might posit that this NC/NA state is associated with decreased activity levels in the areas that subserve both cognition and affect. Newberg et al. have long postulated a relationship with the parietal lobe (Newberg and Iversen, 2003). The PSPL is heavily involved in the analysis and integration of higher-order visual, auditory, and soma-esthetic information (Adair et al., 1995). It is also involved in a complex attentional network that includes the PFC and thalamus (Fernandez-Duque and Posner, 2001). Through the reception of auditory and visual input from the thalamus, the PSPL is able to help generate a three-dimensional image of the body in space, provide a sense of spatial coordinates in which the body is oriented, help distinguish between objects, and exert influences in regard to objects that may be directly grasped and manipulated (Lynch et al., 1980; Mountcastle et al., 1981). These functions of the PSPL might be critical for distinguishing between the self and the external world. It should be noted that a recent study has suggested that the superior temporal lobe may play a more important role in body spatial representation. This has not been confirmed by other reports (Karnath et al., 2001), so the actual relationship between the parietal and temporal lobes in terms of spatial representation remains speculative.

Regardless, deafferentation of these orienting areas of the brain has been suggested as an important mediator in the physiology of meditation (Newberg and Iversen, 2003). We have postulated that the mechanism by which deafferentation might occur is through the action of GABA, released by the reticular nucleus. Thus, GABA, acting as the primary inhibitory neurotransmitter [originally hypothesized by Austin, 1999], might inhibit incoming neuronal information into the PSPL. One can speculate that there is something about certain meditation techniques (NDM) that can trigger this deafferentation effect. If this occurs to a substantial degree it could result in the dampening of cognitive and affective processes creating a state devoid of phenomenological content in which the person may begin to temporarily lose their usual ability to spatially define their notion of self or differentiate the self from the rest of the world—an experience which one could interpret as non-self, or emptiness. Such a notion is supported by clinical findings in patients with parietal lobe damage who have difficulty orienting themselves. The effects of meditation are likely to be more selective and do not destroy the sense of self, but alter the perception of it. This concept of deafferentation of the PSPL has been supported by two imaging studies demonstrating decreased activity in this region during intense meditation (Herzog et al., 1990--1991; Newberg et al., 2001). Other investigators have found support for specific brain functions associated with the non-cognitive, non-affective state. For example, Lehmann et al. (2001) investigated multi-states engendered by a single advanced meditator and found that meditation on the dissolution of the self resulted in increased right brain activity more anterior and superior than other forms of meditation that were visual or mantra dependent: right superior frontal gyrus; right PFC. Hankey (2006) reported psychophysiological correlates for “pure consciousness” associated with TM that were distinctly different than one-pointed and compassion techniques. These changes appear to extend beyond brain processes as Travis and Pearson (2000) reported distinct changes in sympathetic and parasympathetic measures during these states of “pure consciousness.”

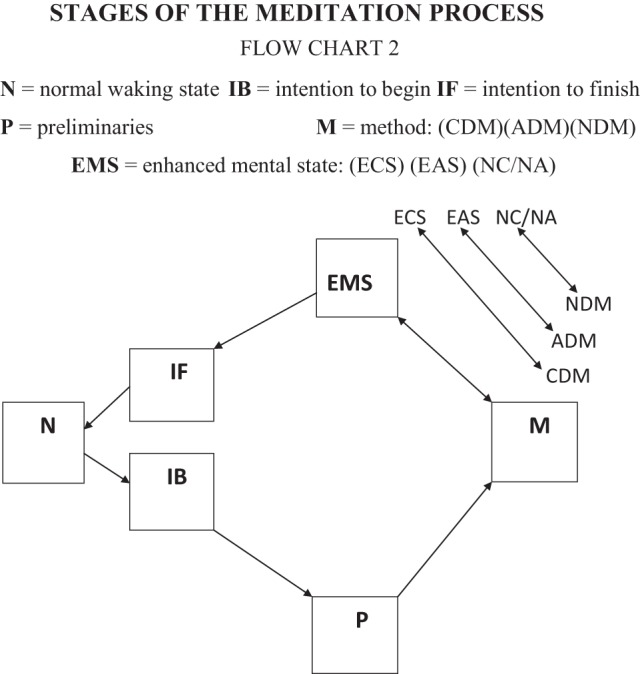



#### The taxonomic keys

Sub-classification of methods within the three Domains is accomplished by applying a system of taxonomic keys, a concept borrowed from the Science of Systematics (Mayr, 1969). We propose that this idea can also be used to address the need for a standardized description of meditation methods (a Standard Profile of Meditation Methods). We argue that this approach enables methods to be more thoroughly and accurately compared. In addition, it allows researchers to account for the neurophysiological effects associated with many of these elements as described in some detail below (for a condensed version of these keys see Table A1 of Taxonomic Keys—Appendix).

***Initial descriptor.*** The initial descriptor should include the name of the technique with reference to any particular style or subset (because several techniques may be grouped together under one generic name, when in fact they may be significantly different). A general reference to the history, origin and culture would be optional.

***The specific keys.*** The keys are based on the explicit directions contained within the method, the overall approach that is employed, and third person elements that can be observed in the laboratory.

1. Description of the specific cognitive strategy(ies) which are prescribed for the practitioner within the method's directions (what one has to do in order to achieve the intended result) i.e., concentration; focused attention or awareness; passive observation without attachment; visualization and imagination; memorization and repetition; selective awareness; effortless awareness; contemplation, introspection, and inquiry; sensual perception(s), etc. Neuroscientifically, this is an important element since there could be distinct changes observed in the brain and body depending on the strategy used. For example, a visualization task is likely to activate the visual cortex, whereas reciting a prayer or phrase is likely to activate the verbal centers of the brain (Newberg et al., 2003, 2010; Peres et al., 2012).
2. Description of the conceptual and/or physical foci—the object(s) of attention i.e., mantra, symbol, image, phrase, idea, narrative, sound, light, etc. Similar to above, the focus on distinct images might produce different physiological effects depending on whether the images are simple, complex, color, or black and white. Listening to music or a voice guiding the meditation might result in activation of the auditory pathways in the brain.
3. Description of any beliefs or special knowledge either suggested or required; i.e., in relation to a particular theoretical, religious, spiritual, metaphysical, or philosophical system. While this is more difficult to identify from a neurophysiological perspective, several studies have explored the neural bases of different beliefs, especially between those who are believers and non-believers (Harris et al., 2009).
4. Notation of whether the eyes are closed or are open and used in some specific fashion. Brain function is clearly affected by visual processes and there is substantial activation in the visual cortex when the eyes are open, especially when observing a complex scene.
5. Notation of whether the process is static or kinetic. “Static” refers to a stationary body but not necessarily an immobile body. Therefore, bodily movements may occur but the body still remains essentially in one place as when the meditator changes postures during a single meditation session (i.e., from an upright sitting position to a more reclined position), or experiences involuntary jerking motions (kriyas). “Kinetic” refers to prescribed movements of the body with specific postural instructions; usually, but not limited to, movement of the extremities such as in walking mindfulness training, Tai Chi, mudras (hand movements), or even “bouncing” as in TM-Siddhi “yogic flying.” This element relates to motor activity in the brain including the motor cortex, basal ganglia, and cerebellum which are all involved in body movement. In addition, movement can be associated with differences in energy utilization, adrenal function, cardiovascular function, and respiratory function.
6. Notation of whether the process is silent or auditory or both. Vocalization will certainly demonstrate specific cortical activity and the auditory cortex and thalamus may be differentially activated in the presence of sound. Regarding silent vs. the vocal use of mantra and chanting, scientists may need to account for the sub-vocalization effect associated with inner speech, even when it is quiet.
7. Notation of whether a specific type of postural position is suggested or required i.e., normal seated, straight spine, lotus position, fully reclined, etc. This key could be considered as a sub-set of the “static” denotation in #5 above. The brain may respond differently to being in, and maintaining, different postures. Proprioceptive functions are likely to be particularly related to this element as the brain works to ensure that a posture is maintained.
8. Notation of whether the process is intrinsic (self-reliant/independent) or extrinsic (dependent on an outside person or process). There is some evidence to suggest that performing meditation under one's own volition vs. being guided can result in substantial differences in brain function. Frontal lobe activity in particular might be affected as evidence suggests decreased frontal activity during externally guided word generation compared to internal or volitional word generation (Crosson et al., 2001). Thus, prefrontal and cingulate activation may be associated with the intrinsic vs. extrinsic aspects of meditation.
9. Notation of whether there are any specific recommendations for type or control of breathing. Breathing, especially when controlled, can result in specific changes in brain and body physiology. Controlled breathing may alter heart rate, blood pressure, and metabolism while also changing the function of the brain (Floyd et al., 2003; Barnes et al., 2008).

Considered in their totality, these taxonomic keys should help to present a relatively full description of the overall meditation technique. Furthermore, since many of these keys can be observed from a third person perspective (i.e., eyes open or closed; body static or kinetic), they provide a more objective way of assessing the method and the extent to which the subject has performed the method.

It should be noted that it has been suggested that some meditation methods could be considered to be essentially “somatic” in nature and therefore warrant classification into a separate Somatic Domain (i.e., Tai Chi, standing Qigong, and some forms of Yoga). However, given that meditation methods are considered to be mental training regimes and not physical training, we concluded that it would be inappropriate to devise an over-arching domain based on somatic considerations. Rather, we account for somatic characteristics as a sub-classification under the fifth (kinetic/static) and seventh (postural) taxonomic keys.

#### Use of the taxonomic keys and domains, nine examples

This section demonstrates how meditation methods can be classified, described, and sub-classified (see Tables 1, 2) using the three Domains and the taxonomic keys. For this demonstration we have selected nine examples (in no particular order) based on the following criteria: fairly well-known Eastern meditation methods which possess historical tradition; meditation methods favored by researchers in recent studies; meditation methods which give the practitioner (and researcher) a reasonable idea of what to expect from a successful meditation session.

**Table 1:** **Examples of the sub-classification of the three domains**.

**CDM**	**ADM**	**NDM**
Samatha	NRLK (dmig med snying rje)	TM
Vipassana		
Kirtan Kriya	Samatha metta and karuna	So'ham Japa
Tai Chi Chuan		

**Table 2:**
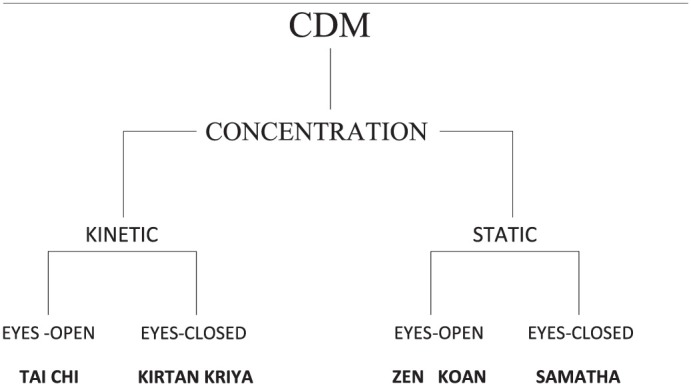
**Sub-classification of four different cognitive-directed methods**.

The following assignment of taxonomic keys and Domains is only intended to demonstrate how the application of this model can be used to more thoroughly describe various meditation methods, and should not be taken to be a final delineation, only a tentative one. Our determinations were based on a review of the relevant literature, personal communication with experienced practitioners, and the personal experience of the authors with several of the following methods. We recognize that this is far from sufficient for the formulation of definitive method profiles and classifications. It would be far better to recruit the input of expert practitioners and attempt to arrive at a consensus regarding the keys and the assignment of Domain. In addition, “final” determinations of Domain would also require confirmation through scientific research (see Suggestions for Future Studies below). For example, in order to make a more definitive assignment of a particular method into the Affective Domain there would first need to be some sort of consensus among experts that the method in question does in fact purport to engender an EAS. Secondly, this claim would need to be supported by research findings of affective phenomenological content and affective neurobiological correlates.

***1. Transcendental meditation (TM).*** Coined and introduced to the West by Maharishi Mahesh Yogi in the late 1950s—early 60s; proponents claim that TM is derived from ancient Indian tradition while others consider it to be based on “a neo-Vedanta metaphysical philosophy” (Olson, 2007).

1. Utilizes awareness, and repetition which proponents claim to be “effortless,” and which induces “automatic self-transcending” to a state of “pure consciousness” (Travis and Shear, 2010a,b; Travis, 2013, pers. commun.).
2. Attention is on a mantra, although proponents state that this is not a form of focused attention, but rather an awareness of mantra (Travis, 2013, pers. commun.).
3. There are no religious/belief requirements.
4. The eyes are closed.
5. The basic form is static; and there is a more advanced kinetic form known as Siddhi “Yogic –flying.”
6. Non-verbal, whereas the mantra is repeated silently, not out-loud.
7. Seated comfortably, no strict postural requirements.
8. Intrinsic.
9. Normal breathing, no special breathing instructions or requirements.

We have classified TM into the **NDM** Domain because the technique as we understand it aims to engender a non-cognitive/non-affective EMS.

***2. Tai Chi Chuan.***^b^ A technique of Chinese Taoist origin has evolved into many different styles; originally developed as a form of martial art (literally translated as grand ultimate fist, and it was known as a boxing style); today is rarely used as a self-defense technique but rather is considered by many to be a form of “movement meditation” (Ospina et al., 2007) for the promotion of good health.

1. Utilizes focused concentration, memorization, and visualization.
2. The physical focus is on the body, the conceptual focus is on the “chi.”
3. Understanding of, and belief in the Taoist chi energy system is suggested but not required, knowledge of the founder and the lineage of the particular style is also encouraged.
4. Eyes are opened and the gaze is used to “lead” the bodily movements.
5. Kinetic.
6. Usually non-verbal.
7. Specific postural forms are required.
8. Usually learned in an extrinsic manner (guidance by an instructor) but progresses to an intrinsic, self-guided practice.
9. Control of breathing may or may not be required.

We have classified Tai Chi into the **CDM** Domain because the technique as we understand it aims to produce a highly concentrative EMS.

***3. Vipassana Meditation.*** A traditional Buddhist technique; Pali/Sanskrit language commonly translated as insight or mindfulness. Caution must be exercised when attempting to classify this method because there are several different forms and styles in current practice all under the same name of Vipassana. For the purposes of this paper we will consider the Goenka method (Goenka, 1987) since many research studies use subjects who employ this technique.

1. Utilizes mindfulness, detached observation, contemplation, and insight.
2. The physical foci of attention is on the body using a “body-scan” technique with a prescribed narrative.
3. Knowledge and belief in the teachings of Buddhism is essential in order to relate the experiences of the meditation to an insight/wisdom into your own nature and to develop an experiential understanding of the “Three Universal Characteristics” (see The Discourse Summaries, Goenka, 1987).
4. Eyes are closed.
5. Static.
6. Non-verbal.
7. Upright seated position on the floor, lotus posture if possible.
8. Intrinsic.
9. Normal breathing, no special breathing instructions or requirements.

We have classified Goenka Vipassana into the **CDM** Domain because the technique as we understand it aims to lead the meditator to an understanding/insight of essential Buddhist principles via a cognitive EMS.

***4. Tibetan Non-Referential Unconditional Loving-Kindness technique (NRLK) (dmigs med snying rje).*** Of traditional Tibetan Buddhist origin.

1. Utilizes concentration, contemplation, and visualization.
2. The conceptual foci of attention is on a prescribed narrative and the emotions that are generated.
3. Understanding and belief in the teachings of Buddhism is suggested but not required, belief in the value of extending loving-kindness to all sentient beings is required.
4. Eyes-open.
5. Static.
6. Non-verbal.
7. Upright seated position, lotus posture if possible.
8. Intrinsic.
9. Normal breathing, no special breathing instructions, or requirements.

We have classified NRLK into the **ADM** Domain because the technique as we understand it aims to create an affective EMS.

***5. Samatha Meditation.*** An ancient contemplative practice believed to have originated in India, traditionally associated with Buddhism, of Pali/Sanskrit language translated as “calm” or “quiescence” but usually considered to mean concentration. There are several forms of samatha meditation and many different objects of focus (forty are listed in traditional Buddhist texts), but all are intended to calm and focus the mind. Here we will consider only two of them.

- **Concentration on the breath**.
  1. Utilizes concentration and visualization as the main cognitive strategies.
  2. The physical focus of attention is on the breath and the parts of the body associated with breathing, especially the nose.
  3. Knowledge and belief in the teachings of Buddhism is recommended but not essential.
  4. Eyes closed.
  5. Static.
  6. Non-verbal.
  7. Seated with straight spine.
  8. Intrinsic.
  9. There are specific breathing instructions.
  We have classified *samatha* concentration on the breath into the **CDM** Domain because the technique as we understand it aims for the practitioner to attain a one-pointed, cognitive EMS.
- **Metta (loving-kindness) and karuna (compassion)**.
  1. Utilizes concentration and visualization as the main cognitive strategies.
  2. The conceptual foci of attention is on a prescribed set of instructions and the emotions that are generated.
  3. Knowledge and belief in the teachings of Buddhism is suggested but not required, belief in the value of extending compassion and loving-kindness to all sentient beings is required.
  4. Eyes closed.
  5. Static.
  6. Non-verbal.
  7. Seated with straight spine.
  8. Intrinsic.
  9. No special breathing instructions or requirements.
  We have classified *samatha metta* and *karuna* meditation into the **ADM** Domain because the technique as we understand it aims to create an affective EMS.

***6. Zen Meditation (zazen).*** Translated from the Japanese as “seated meditation,” of traditional Buddhist Mahayana tradition introduced to Japan in the 11–12th Century. Today there are various schools in the East and West, such as Rinzai and Soto, and there are various techniques, some of which are reserved for more advanced and experienced practitioners. For the purposes of this essay we have chosen an elementary form of the kufu-zazen koan technique used in the Rinzai approach.

1. Utilizes concentration, memorization and repetition, analysis and insight as the primary cognitive strategies.
2. The conceptual focus is on various *koans* (paradoxical riddles).
3. Usually no particular knowledge or belief is required for beginners, knowledge and belief in Buddhist teachings is essential for advancement.
4. Eyes-open.
5. Static.
6. Non-verbal.
7. Specific instructions for seated posture, traditionally with cushion on the floor.
8. Intrinsic and/or extrinsic if there is a Master-student interaction(*sanshi-manbo*).
9. Specific breathing instructions may be applied.

We have classified this particular Zen technique into the **CDM** Domain because, according to our understanding, it aims to lead the practitioner to insight through inquiry. Other Zen techniques directed toward the attainment of one-pointedness would also be classified as CDM; and if an experienced practitioner uses a Zen technique to attain satori it would be considered a NDM.

***7. So'ham Japa (also known as Hamsa).*** Considered to be a Yoga technique of Indian origin, and introduced to the West by Swami Muktananda in the late 1960's—early 1970's.

1. A concentrative strategy.
2. The focus is on mantra and breathing.
3. Belief in the basic teachings and tenants of Hindu philosophy such as Vedanta, and Kashmir Shaivism is suggested but not required; in addition, there is often a strong belief in the power of the Guru-disciple relationship and that the mantra and the technique must be empowered by a Spiritual Master in order to be efficacious and to facilitate a spiritual “awakening” (Muktananda, 1969; Shankarananda, 2003).
4. Eyes are closed.
5. Static.
6. Non-verbal.
7. Comfortable seated posture is suggested.
8. Intrinsic.
9. Specific instructions for awareness and method of breathing.

We have classified So'ham Japa into the **NDM** Domain because the technique, as we understand it, aims at producing a non-cognitive/non-affective EMS, a state of samadhi in which the meditator experiences his “true nature” (Muktananda, 1969).

***8. Kirtan Kriya.*** Is a Yogic practice introduced to the West by Yogi Bhajan in the late 1960's—early 70's. Proponents claim it to be a traditional form of Kundalini Yoga of Northern Indian origin, and taught by a lineage of Sikh Masters for over 500 years (see Kundalini Yoga websites www.3ho.org and www.kundaliniresearchinstitute.org). There are many forms of Kirtan Kriya meditation. The following application of the taxonomic keys is based on the “12 minute” form studied in recent neuroscientific investigations (Khalsa et al., 2009; Wang et al., 2011; Black et al., 2013).

1. Utilizes concentration, memorization, repetition, and visualization.
2. Conceptual focus is primarily on mantra and breath.
3. Knowledge and belief in the basic tenants of Hindu philosophy, and the notions of kundalini energy and chakras, is suggested but not required.
4. Eyes closed.
5. Static in overall body position but also kinetic during those times when the practitioner employs prescribed hand movements known as mudras^c^.
6. Uses both verbal chanting and non-verbal recitation of a series of four mantras^c^.
7. Seated on a chair or on the floor with straight spine.
8. Intrinsic, although extrinsic guided-mediations are also used at times^c^.
9. Normal comfortable breathing, although some practitioners use various forms of pranayam breathing techniques^c^.

We have classified this style of Kirtan Kriya into the **CDM** Domain because the technique, as we understand it, aims at calming and focusing the mind by producing a cognitive EMS.

## Summary

This paper has attempted to be helpful to researchers and writers in the field of contemplative neuroscience in several ways. In order to improve communication among scholars, we proposed a conceptual model of meditation as a dynamic process that could help to avoid the conflation of definition that has plagued this field for many years. Considering meditation as a series of connected yet distinct stages also presents new opportunities for targeted research and evaluation.

Our effort was also motivated by the belief that in order for this (or any) relatively new field to progress it is essential for researchers to have a valid, reliable, and universally acceptable means with which to taxonomize and compare their findings. To this end we have attempted to influence due consideration toward the utilization of a third-person codification for meditation methods, for which we chose the domains of Affect and Cognition. In addition, we have attempted to demonstrate the advantages of utilizing an essentialist-type taxonomic system to identify the functional essence of methods in terms of their immediate intended goal, which we labeled “directionality.” This approach enabled us to formulate three overarching, potentially orthogonal categories, and segregate meditation methods into three Linnaean-type Domains: CDM, ADM, and NDM. We also used this nomenclature to describe the causally related enhanced states associated with each of these Domains.

Finally, we attempted to address the current lack of a standardized descriptive protocol for the study of meditation methods, which we feel is an important oversight and impediment. We proposed the use of a taxonomic key system as a means of delineating the salient features into a Standard Profile of Meditation Methods. We also provided examples of how such a protocol could be used to describe several well-known and often studied methods, and how it could be used to sub-classify methods within a given Domain.

## Suggestions for future study

- The use of a system of validated taxonomic keys to create definitive descriptive profiles, sorted by Domain, that could serve as a comprehensive compendium of meditation methods. This can be accomplished by soliciting the input of expert practitioners and proponents of each method (similar to the approach used by Koshikawa and Ichii (1996) and by using a questionnaire and standardized interview methodology similar to the modified Delphi consensus technique employed by Ospina et al. (2007) and Bond et al. (2009).
- Research studies utilizing a variety of established neuroscientific methods to test the usefulness and orthogonal relationship of the various stages of the proposed process model.
- Research studies utilizing a variety of established neuroscientific methods to test the validity of our three Domains based on affective, cognitive, and null enhanced states. The hope would be to develop a neurophysiological “signature” or profile for each Domain. There is support for this notion that a given EMS can be identified in the laboratory setting using standardized methods (Travis and Pearson, 2000; Travis et al., 2002; Travis and Arenander, 2006; Newberg and d'Aquili, 2001; Lutz et al., 2007). We posit that the three types of EMS proposed in this paper can be similarly defined using the methodologies that have been previously developed and tested.
- Developing the methodology and instrumentation for monitoring and measuring kinetic forms of meditation, as well as devising additional taxonomic keys for these types of methods.

## Conclusion

Our proposal for a new taxonomy of meditation is intended to offer a fresh approach to this difficult but necessary task, and hopefully will help to further the process for developing a much needed standard. It is not to be construed that we are here claiming to have arrived at a complete and final explication. Rather we have offered an alternative paradigm with the intention of stimulating interest in the advantages of using an essentialist third-person approach. Should this model prove useful to the current field of contemplative neuroscience, we fully expect it to be tested and challenged by future findings and new theories. Even the “Mother” of all taxonomies, the great work of Linnaeas (which has been a standard for almost 300 years), has become somewhat obsolete given recent findings from cellular and genetic biology (Ereshefsky, 2000).

Our three overarching Domains are intended to provide researchers with a reliable conceptual framework for their findings by pointing to cognitive and affective states and their neurobiological correlates. We contend that it is essential for researchers (and all interested parties) to know more than just the name and a general description of the particular meditation method that is being studied. The use of the taxonomic keys in conjunction with the three Domains presents a replicable descriptive standard for the study of meditation methods. This protocol offers a way for researchers to more effectively account for the neurobiological correlates associated with those salient features of the method that could confound their findings. Undoubtedly, research findings are affected by factors such as whether the subject is meditating with their eyes open or shut, using specific body movements, or verbalizing in some manner. Such information, when presented in a standardized fashion, could facilitate a more cogent analysis of the differences and similarities reported for various meditation methods, and help with the task of trying to isolate the state from the method.

We recognize that this proposal, as with any taxonomy, must ultimately be evaluated by its usefulness to scholars and researchers. As such, we have purposely steered clear of metaphysically charged claims of attainment and non-conceptual realms commonly associated with contemplative practices and beliefs, since such considerations are currently outside of the scope of scientific measurement and analysis. This is not a value judgment, but simply a matter of practicality. We do not dismiss or discount such phenomena as “enlightenment” or the role of the Guru-disciple relationship in the process of “awakening”; we simply have chosen to focus on the more tangible aspects of meditation practice.

We fully expect that as we learn more about the phenomenon of meditation, the assumptions and structure of our definitional and taxonomic models will need to be modified or replaced to adapt to new understandings. Hopefully researchers will be interested in our proposals, will test their efficacy, and offer suggestions and improvements that will ultimately lead to the attainment of a consensual definition and taxonomy for the field of meditation research.

### Conflict of interest statement

The authors declare that the research was conducted in the absence of any commercial or financial relationships that could be construed as a potential conflict of interest.

## Footnotes

^a^It is important to note that we are not assuming that these three states are in some way totally “pure” and unrelated to each other, but rather that they are essentially different. For example, we are not saying that an ECS needs to be totally and completely devoid of any affective phenomenological content, and vice versa. Rather, we are proposing that each of these categories represents a specific and distinctive type of experience that allows each one to be differentiated from the others.

^b^We chose to include Tai Chi on this list because it is a good example of a kinetic form, even though the field currently does not have unobtrusive instrumentation with which to evaluate such a method and it is still debatable whether it is truly a form of meditation or not.

^c^The Kirtan Kriya method poses descriptive, taxonomic, and research challenges because there are options for applying several opposing keys during a single meditation session. As such, in this case, and in other similar cases, it is important to note which options are, and are not, being employed by the practitioner at any given point in time.

## Appendix

**Table A1:** **The taxonomic keys**.

The **initial descriptor** should include the name of the method including mention of any particular style or subset (because several techniques may be grouped together under one generic name, when in fact they may be significantly different). A general reference to the history, origin and culture is optional.
**THE SPECIFIC KEYS**
(1) Description of the specific cognitive strategy(ies) which are prescribed for the practitioner within the method's directions (what one has to do in order to achieve the intended result) i.e., concentration; focused attention or awareness; passive observation without attachment; visualization and imagination; memorization and repetition; selective awareness; effortless awareness; contemplation, introspection and inquiry; sensual perception(s), etc.
(2) The conceptual and/or physical foci—the object(s) of attention i.e., mantra, symbol, image, phrase, idea, narrative, sound, light, etc.
(3) Any beliefs or special knowledge either suggested or required i.e., a particular theoretical, religious, spiritual, metaphysical, or philosophical system
(4) Whether the eyes are closed or are open and used in some specific fashion
(5) Whether the process is static^*^ or kinetic^**^
(6) Whether the process is non-verbal (silent) or verbal (auditory) or both
(7) Whether a specific seated or reclined postural form is suggested or required
(8) Whether the process is intrinsic (self-reliant/independent) or extrinsic (dependent on an outside person or process)
(9) Whether there are any specific recommendations for type or control of breathing

* “static”: refers to a stationary body but not necessarily an immobile body. Therefore, bodily movements may occur but the body still remains essentially in one place as when the meditator changes postures during a single meditation session (i.e., from an upright sitting position to a more reclined position), or experiences involuntary jerking motions (kriyas). Specific postural criteria may or may not be present.

** “kinetic”: refers to prescribed movements of the body with specific postural instructions; usually, but not limited to, movement of the extremities such as in walking mindfulness training, Tai Chi, mudras (hand movements), or even “bouncing” as in TM-Siddhi “yogic flying.”

## End notes

1. “pure consciousness”:
  “…. ‘pure *consciousness*’ or *‘emptiness’* is devoid of phenomenological content, and by its nature requires transcending of the processes and objects of meditation. If processes or objects were there, it would not be this widely described experience.” Travis and Shear (2010b) It “is ‘pure’ in the sense that it is free from the processes and contents of knowing. It is a state of ‘consciousness’ in that the knower is conscious through the experience, and can, afterwards, describe it. The ‘content’ of pure consciousness is self-awareness. In contrast, the contents of normal waking experiences are outer objects or inner thoughts and feelings.” (p. 79). “Subjectively, this state is characterized by the absence of the very framework (time, space, and body sense) and content (qualities of inner and outer perception) that define waking experiences. Physiologically, this state is distinguished by the presence of apneustic breathing, autonomic orienting at the onset of breath changes, and increases in the frequency of peak EEG power.” Travis and Pearson (2000, p. 77).
2. “absolute unitary being” (AUB):
  “…. an absolute sense of unity — without thought, without words, and without sensation …without ego in a state of pure, undifferentiated awareness.” Newberg and d'Aquili; (2001, p. 119–27).
3. “non-dual awareness” (NDA):
  There is “a more profound difference, one that cannot be adequately captured within a single-dimension characterization of attentional strategy. Both focused attention and open monitoring styles of meditation contain an essentially dualistic orientation of ‘subject-observing-object’. there is another group of meditations that do not employ this strategy, but instead rely on accessing a level of awareness that is inherently free from this dualistic subject-object construct. This non-conceptual awareness has sometimes been termed non-dual awareness, open awareness or open presence.” Josipovic (2010).
  “There are 2 aspects of non-duality: (1) non-duality without experiential content, also known as ‘pure consciousness’ in Vedanta or ‘isolated clear light’ in Tibetan Buddhism; which is Absolute only and (2) non-duality with full experiential content; a.k.a ‘open presence’ in Tibetan Buddhism, Saguna Brahman of Vedanta, etc., in other words the unity of Absolute and Relative.” Josipovic (2013, pers. commun.).
4. “focused attention” (FA):
  “A widespread style of Buddhist practice consists in sustaining selective attention moment by moment on a chosen object, such as a subset of localized sensations caused by respiration. To sustain this focus, the meditator must also constantly monitor the quality of attention. … while cultivating the acuity and stability of sustained attention on a chosen object, this practice also develops three skills regulative of attention: the first is the monitoring faculty that remains vigilant to distractions without destabilizing the intended focus. The next skill is the ability to disengage from a distracting object without further involvement. The last consists in the ability to redirect focus promptly to the chosen object.” Lutz et al. (2008b, p. 163–169).
5. “open-monitoring” (OM):
  “Open monitoring or mindfulness-based meditations, involve the non-reactive monitoring of the content of ongoing experience, primarily as a means to become reflectively aware of the nature of emotional and cognitive patterns.” Raffone and Srinivasan (2010). “Open monitoring practices are based on an attentive set characterized by an open presence and a non-judgmental awareness of sensory, cognitive and affective fields of experience in the present moment and involves a higher-order meta-awareness of ongoing mental processes” (Cahn and Polich, 2006)—in Travis and Shear (2010a).
  “While varied, OM practices share a number of core features, including especially the initial use of FA training to calm the mind and reduce distractions. As FA advances, the well developed monitoring skill becomes the main point of transition into OM practice. One aims to remain only in the monitoring state, attentive moment by moment to anything that occurs in experience without focusing on any explicit object. To reach this state, the practitioner gradually reduces the focus on an explicit object in FA, and the monitoring faculty is correspondingly emphasized. Usually there is also an increasing emphasis on cultivating a ‘reflexive’ awareness that grants one greater access to the rich features of each experience, such as the degree of phenomenal intensity, the emotional tone, and the active cognitive schema.” Lutz et al. (2008b, p. 163–169).
6. “open-presence” (OP):
  “… unlike other meditations, at advanced stages of the practice there is no attempt either to suppress or to cultivate any particular mental content. One does not focus, for example, on a visualized image or on a sensory object such as a sensation made by the breath. In this sense the state of Open Presence is objectless. Nevertheless, even though higher levels of the practice do not involve any particular content or object, it also is important for content to be occurring in the mind because to cultivate an awareness of the invariant nature of experience, one must be having experiences. Indeed, for beginners it is preferable that the experiences be especially striking or clear. Thus, even though the meditation is objectless, it is not a state of blankness or withdrawal. Sensory events are still experienced, sometimes even more vividly. In terms of technique, this facet of the meditation is indicated by the fact that one meditates with the eyes open and directed somewhat upward.” “The move to an emphasis on subjectivity is further encouraged by dropping any deliberate focus on an object. As a sensory content or mental event occurs, one observes it (sometimes along with the momentary use of a discursive strategy), and then one releases any focus on it. This is similar to the Vipassanâ practice discussed above, except that after releasing the content or event one does not return to any object. Instead, one releases the mind into its ‘natural state’ (rang babs), which one understands to be the state reflecting only the invariant nature of consciousness, and not the accidental properties of subject and object.” Lutz et al. (2007, p. 38–42).
