# Corrigendum: Toward a unifying taxonomy and definition for meditation

**DOI:** 10.3389/fpsyg.2019.02206

**Published:** 2019-10-09

**Authors:** Jonathan D. Nash, Andrew Newberg, Bhuvanesh Awasthi

**Affiliations:** ^1^Chiangmai, Thailand; ^2^Myrna Brind Center for Integrative Medicine, Thomas Jefferson University Hospital and Medical College, Philadelphia, PA, USA; ^3^Waisman Center, University of Wisconsin-Madison, Madison, WI, USA

**Keywords:** meditation, taxonomy, definition, contemplative traditions, contemplative neuroscience, cognition, affect, consciousness

Bhuvanesh Awasthi was not included as an author in the published article. He provided input during the initial preparation, research material and drafting of the manuscript. The author list has now been updated accordingly.

The authors apologize for this error and state that this does not change the scientific conclusions of the article in any way. The original article has been updated.

